# Sustainable planning in Wuhan City during COVID-19: an analysis of influential factors, risk profiles, and clustered patterns

**DOI:** 10.3389/fpubh.2023.1241029

**Published:** 2023-12-13

**Authors:** Peng Zhou, Hailu Zhang, Lanjun Liu, Yue Pan, Yating Liu, Xuanhao Sang, Chaoqun Liu, Zixuan Chen

**Affiliations:** School of Civil Engineering and Architecture, Wuhan Institute of Technology, Wuhan, China

**Keywords:** Wuhan city, random forest, spatial analysis, sustainable planning, machine learning

## Abstract

The outbreak of novel coronavirus pneumonia (COVID-19) is closely related to the intra-urban environment. It is important to understand the influence mechanism and risk characteristics of urban environment on infectious diseases from the perspective of urban environment composition. In this study, we used python to collect Sina Weibo help data as well as urban multivariate big data, and The random forest model was used to measure the contribution of each influential factor within to the COVID-19 outbreak. A comprehensive risk evaluation system from the perspective of urban environment was constructed, and the entropy weighting method was used to produce the weights of various types of risks, generate the specific values of the four types of risks, and obtain the four levels of comprehensive risk zones through the K-MEANS clustering of Wuhan’s central urban area for zoning planning. Based on the results, we found: ①the five most significant indicators contributing to the risk of the Wuhan COVID-19 outbreak were Road Network Density, Shopping Mall Density, Public Transport Density, Educational Facility Density, Bank Density. Floor Area Ration, Poi Functional Mix ②After streamlining five indicators such as Proportion of Aged Population, Tertiary Hospital Density, Open Space Density, Night-time Light Intensity, Number of Beds Available in Designated Hospitals, the prediction accuracy of the random forest model was the highest. ③The spatial characteristics of the four categories of new crown epidemic risk, namely transmission risk, exposure risk, susceptibility risk and Risk of Scarcity of Medical Resources, were highly differentiated, and a four-level integrated risk zone was obtained by K-MEANS clustering. Its distribution pattern was in the form of “multicenter-periphery” gradient diffusion. For the risk composition of the four-level comprehensive zones combined with the internal characteristics of the urban environment in specific zones to develop differentiated control strategies. Targeted policies were then devised for each partition, offering a practical advantage over singular COVID-19 impact factor analyses. This methodology, beneficial for future public health crises, enables the swift identification of unique risk profiles in different partitions, streamlining the formulation of precise policies. The overarching goal is to maintain regular social development, harmonizing preventive measures and economic efforts.

## 1 Introduction

Since its discovery in Wuhan in December 2019, the COVID-19 has caused 400 million infections and 5.76 million deaths worldwide, with 140,000 infections and 5,700 deaths in China as of February 9, 2022. The disease is mainly transmitted from patients and asymptomatic infected persons to susceptible people by droplet or contact transmission and can cause damage to the human lungs and sequelae (1). Both developed countries and developing countries have been severely affected (2). Even in developed countries, the epidemic control has experienced multiple outbreaks to varying degrees. Balancing the relationship between the economy and epidemic prevention is the focus of epidemic prevention work. When formulating epidemic prevention policies (3). Governments of various countries have formulated various protection policies for different industries, especially the consumer industry (4). People in developing countries are more seriously affected by the epidemic. The epidemic prevention and control in developing countries is even more severe, medical resources are scarce, and supporting public services are not perfect. In the context of the global epidemic, import and export trade has been seriously affected (5). Many developing countries severely impacted (6). Due to institutional policy reasons, some socialist developing countries have concentrated and dispatched resources quickly, which is relatively timely for epidemic prevention and control (7). However, the balance between urban regional economy and epidemic prevention is still worth exploring.

Epidemic prevention and control have been liberalized, and the epidemic is spreading rapidly in densely populated urban areas with a trend toward multiple waves of mass infection, causing varying degrees of impact on healthcare systems around the country (8). Studying the risk of this virus in various regions of cities is crucial for urban outbreak prediction and the timely deployment of medical resources (9). It helps formulate targeted policies for different regions and provides scientific basis for how to balance economy and prevention and control.

Current COVID-19 urban risk studies primarily focus on macro-scale assessment, measuring transmission risk at national, regional, or provincial scales from a city perspective, using population migration and intercity infection cases as main risk quantifiers (10).Global research often approaches from the perspectives of population characteristics and urban environments (11). For instance, there are studies involving multivariate regression analysis of full-scale population characteristics and COVID-19 cases in the United States (12), multivariate Pearson correlation analysis and inverse distance weighted regression of COVID-19 cases in Colorado (13), and cluster analysis of factors affecting the COVID-19 epidemic in New York (14). In Italy, multivariate analyses have been conducted on sociodemographic, healthcare, and transmission factors within a certain range (15). Through spatial correlation analysis, it has been found that the incidence of COVID-19 in Germany is related to socio-economic factors, population characteristics, and environmental composition (17).

Research related to China often operates on a smaller scale, as the formulation of prevention and control policies is typically done at the provincial level. Therefore, the research perspective tends to focus more on the state of the epidemic at the provincial level (18). Epidemic-related research found that cases in Hubei Province were concentrated in central areas such as Wuhan, Ezhou, and Xiaogan (19). In other provinces, such as Jiangxi Province, the spatial spread of the epidemic is in the form of a “far Hubei single-core” structure form (20). In the microscopic space inside the city, the spread and risk of the COVID-19 epidemic is still the most important part of public safety and health, so it is indispensable to study the microscopic risk of COVID-19 (21). Focusing on the spread of COVID-19 in Wuhan, it was found that the density of tertiary hospitals, commercial density, subway station density, construction scale, aging population, and mixed land use had a significant impact on the epidemic (22). Relevant studies have found that the older adult population accounts for more than half of the total number of Weibo help seekers. Moreover, a close correlation between the two was also found in the spatial distribution characteristics, confirming that the older adult population is a high-risk and high-prevalence population for group COVID-19 outbreaks (23).

Regarding the research methods for urban risk, current studies mostly use geo-graphically weighted regression (24), spatial heterogeneity analysis (25), spatiotemporal aggregation analysis, and factor analysis (26, 27). This study employs innovative techniques for gathering COVID-19 patient locations and demographic/economic data in Wuhan City. It evaluates transmission, exposure, susceptibility, and healthcare resource scarcity risks using empirical Bayesian kriging interpolation, the Huff gravity model, and K-means clustering. It establishes a microscale epidemic risk assessment index system, aiming to comprehensively analyze early-stage epidemic risks in Wuhan, including transmission, exposure, population susceptibility, and medical resource scarcity, from an intracity population-economic perspective. The findings offer insights to assist the government in adopting rational prevention and control methods, mitigating epidemic impact, and formulating targeted, science-based prevention and control strategies, thereby balancing epidemic prevention and economic development.

## 2 Materials and methods

### 2.1 Study area

**Figure 1:**
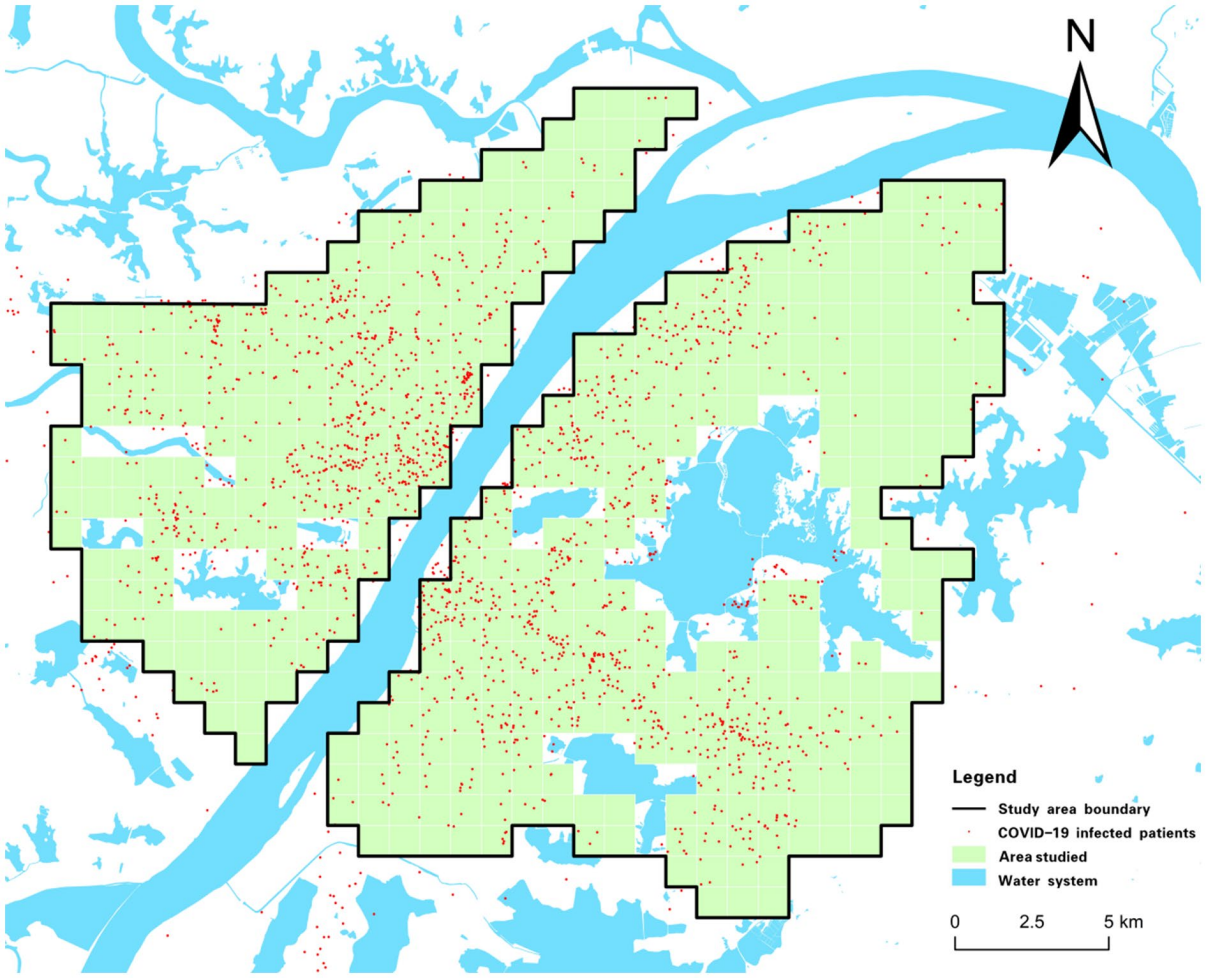
Overview of the study area.

This study selects the planned central urban area of Wuhan as the research object (Figure 1). The area covers 609 km^2^ and seven administrative jurisdictions, including Hankou District, Jiang’an District, and Qingshan District, which are distributed on both sides of the Yangtze River and differ in regional patterns, spatial forms, and socioeconomic levels (28). Wuhan has many lakes, among which Wuchang District, Hongshan District, and Qingshan District have a large area of water. Wuchang District is an old urban area with a long history in Wuhan. Hongshan District is part of the Wuchang District that is developed to the east, and the main part of Qingshan District is developed by the Wuhan Iron and Steel Group and its affiliated residential areas. Hanyang District is located at the confluence of the Yangtze River and Hanshui River, with undulating terrain and a large lake area. It was originally an industrial base of Wuhan, where the economic development level is general (29).

### 2.2 Data sources

Wuhan, as the initial epicenter of the COVID-19 pandemic, was the first city worldwide to grapple with the outbreak. The high mortality rate associated with the virus, coupled with a dearth of medical resources, necessitated the enforcement of stringent lockdown measures. Infected individuals were confronted with a myriad of unanticipated issues, and the pursuit of offline assistance was obstructed, if not entirely precluded, due to resource deficiencies among other impediments. As a result, a significant proportion of patients requiring assistance resorted to social media platforms. Sina Weibo, with its extensive user base exceeding 516 million active users monthly, emerged as the predominant platform for soliciting help. Throughout the lockdown period, there was a substantial influx of help-seeking posts originating from Wuhan on Weibo, thereby providing data that is somewhat representative of infection cases (30). The location data for these cases were extracted from Sina Weibo’s help-seeking data using a Python software crawler for each district. Public photographs of infection cases across various regions were authenticated, followed by an exhaustive analysis of over 600 timestamped images from each district. The spatial data pertaining to infection cases were rendered accessible and precise through the verification and modification of official announcement data, Sina Weibo help data, and public photo data from each district. The specific data sources are shown in Table 1. In this study, the main urban area of Wuhan was taken as the study area and gridded, and data on the population, GDP, POI, and COVID-19 cases in the area were collected. Kernel density analysis and empirical Bayesian kriging interpolation were used to derive the transmission, exposure, susceptibility, and medical resource scarcity risks in the study area. To develop better epidemic prevention and control strategies, we used the random forest algorithm to explore the contribution of influencing factors, and used the K-means algorithm to divide the study area into four zones with different risk characteristics and developed respective epidemic prevention strategies.

**Table 1:** Data source.

Data	Source
Population data, gross domestic product (GDP), and nighttime lighting data	Resources, Environment and Resources Science Data Center (https://www.resdc.cn)
House prices in residential areas of various POI points of interest (subway stations, shopping malls, tertiary hospitals, hospitals, etc.)	Baidu map (https://map.baidu.com)
Wuhan designated hospital data and community publicity photos during the epidemic	Wuhan Health Commission website (http://wjw.wuhan.gov.cn).
Water system, building data, and road network data	Open street map (https://www.openstreetmap.org/)
COVID-19 epidemic case help-seeking data	Sina Weibo (https://www.weibo.com/)

### 2.3 Research methodology

#### 2.3.1 Risk assessment index system construction

**Figure 2:**
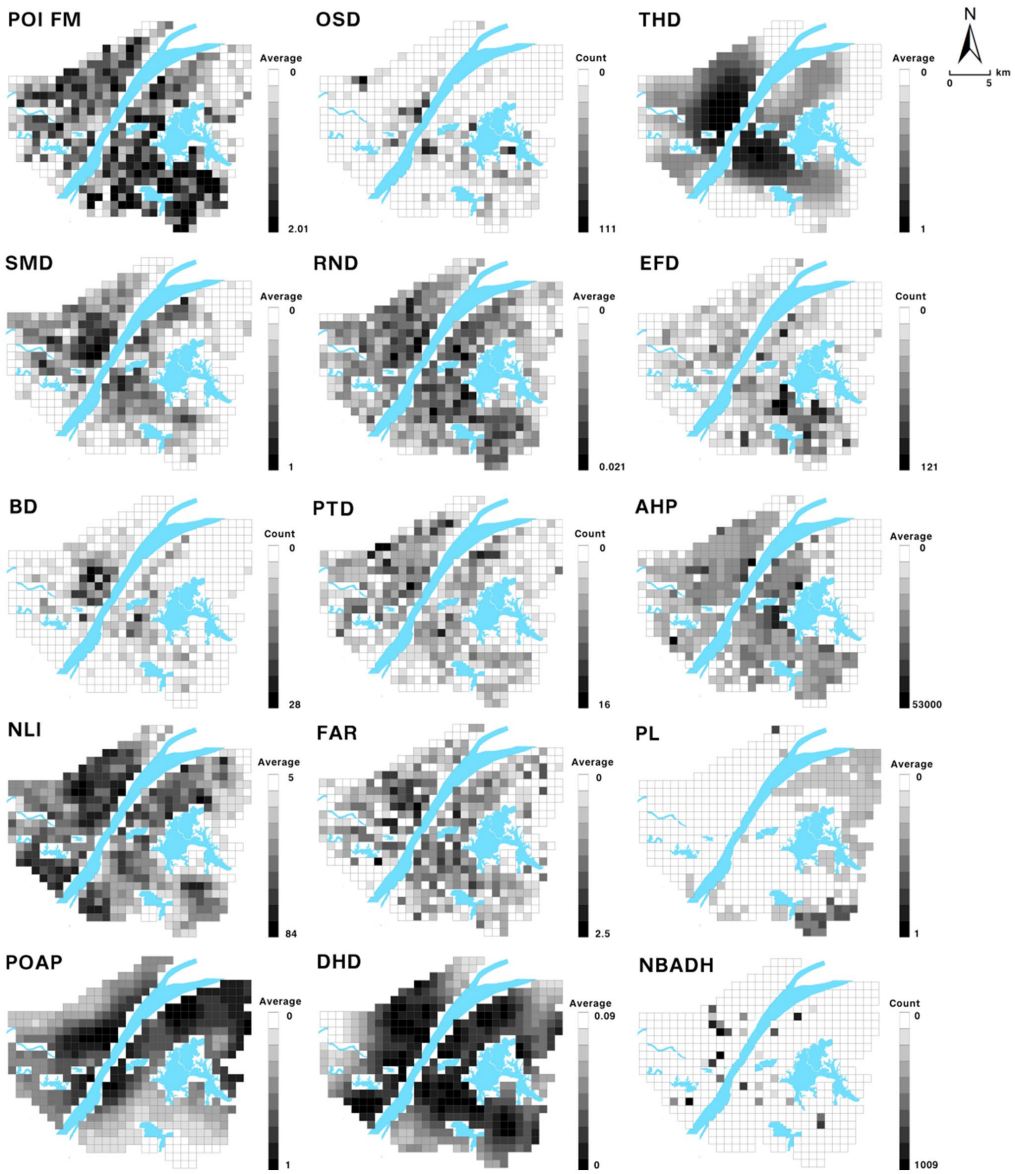
Evaluation parameter visualization.

First, the study area was cut into 1 km × 1 km grids and 437 spatial grids were obtained by sieving parts of the Yangtze River, East Lake, and other water bodies with large areas. Subsequently, various types of raw Wuhan data were processed and connected to 437 grids using raster turning points and spatial connections to form the spatial data. The risk types, risk meanings, assessment parameters, and operation methods of each risk were summarized through relevant literature (31). Four types of epidemic risks were constructed by combining various types of Wuhan spatial data, and the risk assessment index system was tabulated and established (Table 2). Figure 2 shows a visualization of the 15 types of assessment parameters (32). In this study, all evaluation parameters transformed from raw data were normalized, that is, each value was subtracted from the minimum of all values and divided by the difference between the maximum and minimum values. Thus, the largest value was assigned 1 and the smallest value was assigned 0 to avoid mutual interference between different parameters in the calculation of risk.

**Table 2:** Risk assessment index system.

Risk type	Risk implications	Evaluation parameters	Operation method
Transmission risk (TR)	The chance that the virus will spread from an infected individual to a susceptible individual	Poi Functional Mix (POI FM)	Raster turning points, spatial connections
		Open Space Density (OSD)	Kernel density analysis, spatial connectivity
		Tertiary Hospital Density (THD)	Kernel density analysis, raster turning points, and spatial connectivity
		Shopping Mall Density (SMD)	Kernel density analysis, spatial connectivity
		Road Network Density (RND)	
		Educational Facility Density (EFD)	
		Bank density (BD)	
		Public Transport Density (PTD)	Kernel density analysis, spatial connectivity
		Average Housing Price (AHP)	Kerriging interpolation, spatial connectivity
Exposure risk (ER)	Risk of potentially coming into contact with the virus and becoming infected	Night-time Light Intensity (NLI)	Space connection
		Floor Area Ration (FAR)	Create buffers, spatial connections
Susceptibility risk (SR)	An individual’s risk of contracting the COVID-19 virus	Poverty Level (PL)	Raster turning points, spatial connections
		Proportion of Aged Population (POAP)	Empirical Bayesian kriging interpolation
Risk of scarcity of medical resources (RSMR)	Availability and adequacy of medical resources	Designated Hospital Distance (DHD)	Huff gravity model
		Number of Beds Available in Designated Hospitals (NBADH)	

#### 2.3.2 Technical methods for indicator processing

##### 2.3.2.1 Kernel density analysis

For the acquired POI point raw data, first, classification is performed to obtain the subway station POI, shopping mall POI, and tertiary hospital POI (16) and so on. Density raster data are derived by the kernel density analysis method, and then raster transfer point and spatial connection tools are used to obtain the density value of each grid. The purpose of kernel density analysis is to obtain an estimate of each point of the density function that can approximate the distribution of the data so that the distribution of the data can be represented more accurately (33).

##### 2.3.2.2 Empirical Bayesian kriging interpolation

For incomplete data, the complete data needs to be predicted by interpolation, for example, older adult population proportion data obtained by empirical Bayesian kriging (EBK) technique interpolation (34). Unlike other kriging methods in ArcGIS that require manual adjustment of parameters to obtain accurate results, it automatically calculates the interpolation parameters through a subset and simulation process. The advantage of the EBK method is that the standard prediction error is more accurate than that of other kriging methods (35). The advantage of the EBK method is that the standard error of prediction is more accurate than that of other kriging methods. In addition, it provides a relatively more accurate prediction for common, non-smooth data, and smaller datasets (36).

##### 2.3.2.3 Huff gravity model

We estimated the accessibility of healthcare facilities using a gravity model that uses the number of beds available as a measure of attractiveness. The gravity model approximates the abundance of healthcare resources around the grid based on the distance between the grid centroid and destination (designated hospitals). The gravity model we used was a transformed form of the Huff gravity model (37) as follows:


(1) $$P_{j}=\overset{n}{\underset{i=1}{\sum }}\frac{w_{i}}{D_{ij}^{a}}$$

Among them, $P_{j}$ = j Abundance of medical resources in the district.

$w_{i}$ = attractiveness of each designated hospital *i*; in this case, the number of beds.

$D_{ij}$ = distance from area *j* to designated hospital *i*.

a = Index applied to the distance, which was taken as 1 in this study.

#### 2.3.3 Random forest model

The random forest model is an integrated learning model based on a combination of multiple decision trees. The method uses bootstrap resampling. It draws several samples with a consistent number of features from the original training set, performs decision tree modeling on each sample, and then combines the predictions of multiple decision trees to derive the final result by voting or taking the average (38).

**Figure 3:**
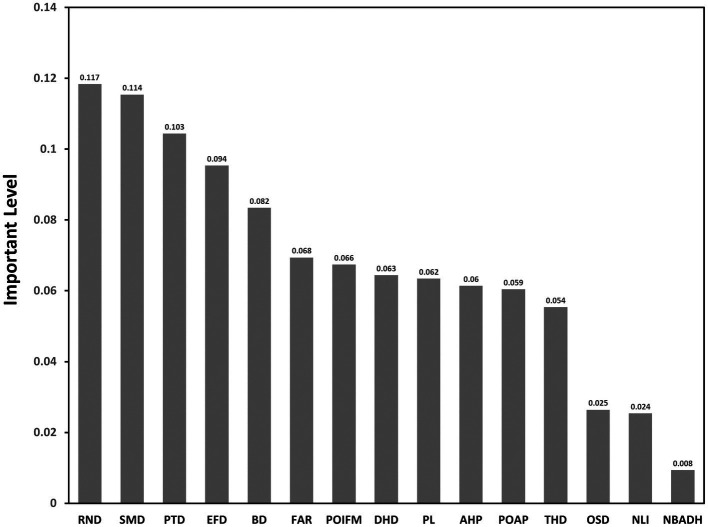
Contribution rate of each indicator factor.

Random forest can analyze the contribution value of the factors affecting the new coronavirus epidemic, and more intuitively display the impact on the distribution of cases. A total of 2,000 samples [1,000 samples with confirmed cases and 1,000 samples without confirmed cases; based on these 2,000 sets of sample data, 70% of the sample points (i.e., 700 sample points with confirmed cases and 700 samples without confirmed cases)] were set to be selected as the training set, and the remaining 30% of sample points were used as the validation set. The remaining 30% of the sample points were used as validation sets. The main parameters of the model are set as follows: *n* estimators = 100, criterion = ‘Gini,’ max_depth = ‘None,’ min_samples_split = 2, min_samples_leaf = 20, max_features = ‘sqrt,’ min_ impurity_decrease = 0.0, bootstrap = True, oob_score = True, n_jobs = 1, random_state = None, and the random forest model was run using python software. The contribution ranking of each indicator factor was obtained (Figure 3).

The random forest model has been applied in many previous studies to evaluate the importance of features, and important weights were derived based on the Gini index. The importance of a feature was determined by calculating the average change in the Gini index for each feature at each decision tree node segmentation and comparing the average change in the Gini index for different features as a percentage of the sum of the average change in the Gini index for all features (39). VIM denotes the feature importance score and GI denotes the Gini index. Suppose there are m features ( $X_{1}$ , and $X_{2}$ , and $X_{3}$ ,...,, and $X_{m}$ ); first, calculate each feature $X_{j}$ of the Gini index score $VIM_{j}^{\left(\mathrm{Gini}\right)}$ which is the j average of the amount of node split impurity change of the first feature in all decision trees. The calculation formula is as follows:


(2) $$GI_{m}=\overset{k}{\underset{k=1}{\sum }}\underset{k\prime }{\sum }p_{mk}p_{mk\prime }=1-\overset{\left|k\right|}{\underset{k=1}{\sum }}p_{mk}^{2}$$

(3) $$VIM_{jm}^{\left(\mathrm{Gini}\right)}=GI_{m}-GI_{l}-GI_{r}$$

where k denotes the number of categories and $p_{mk}$ denotes the category *k* in the node m and the proportion of the nodes. $VIM_{jm}^{\left(\mathrm{Gini}\right)}$ denotes the feature $X_{j}$ in the node m and the amount of change in the Gini index at the time of splitting, $GI_{l}$ and $GI_{r}$ denote the Gini indices of the two new nodes after branching.

If the feature $X_{j}$ in decision tree i , the nodes that appear in the decision tree are the set M , then $X_{j}$ in the first i tree is the importance of


(4) $$VIM_{jm}^{\left(\mathrm{Gini}\right)}=\underset{m\in M}{\sum }VIM_{jm}^{\left(\mathrm{Gini}\right)}$$

If there is a total of n trees, then $X_{j}$ the sum of the Gini index changes in all decision trees, is


(5) $$VIM_{j}^{\left(\mathrm{Gini}\right)}=\overset{n}{\underset{i=1}{\sum }}VIM_{ij}^{\left(\mathrm{Gini}\right)}$$

Finally, it is normalized to obtain the feature $X_{j}$ of importance.


(6) $$VIM_{j}=\frac{VIM_{j}}{\sum_{i=1}^{c}VIM_{i}}$$

Because random forest is an integrated algorithm, its accuracy is higher than that of a single algorithm. Bootstrap resampling involves put-back sampling, which significantly improves the randomness of the training set, making the model less prone to overfitting and more stable. This makes it one of the best machine learning models that is currently recognized (40).

#### 2.3.4 Entropy power method

Entropy was originally a thermodynamic concept in physics and was later introduced into information theory by Shannon, who called it “information entropy.” The basic idea of the entropy method is to determine the weight of each index according to the variability of each variable and then obtain a relatively objective weight through correction. The entropy weight method is widely used in various fields, such as engineering and economics, owing to its broad applicability, high accuracy, and objectivity. Algorithm steps for determining weights using entropy weight method (41).

The entropy weight method sets the initial data matrix of object set *X* as *X* = (*X_ij_*) *n* × *m*, which includes *n* objects to be evaluated and m evaluation indices. The detailed steps for obtaining the weights of the evaluation indices using the entropy weight method are as follows:

Standardization of the initial index data: let the standardized value of each index datum be *Y_ij_*; then, we have:


(7) $$Y_{ij}=\frac{X_{ij}-\min\left(X_{i}\right)}{\max\left(X_{i}\right)-\min\left(X_{i}\right)}$$

Calculate the information entropy value of each indicator: the information entropy value of the data of the *j*th group of indicators is:


(8) $$S=-\ln{\left(n\right)}^{-1}\overset{n}{\underset{i=1}{\sum }}p_{ij}\ln p_{ij}$$

where $p_{ij}=Y_{ij}/\sum_{i=1}^{n}Y_{ij}$ , if $p_{ij}=0$ , then define $\lim_{p_{ij}\to 0}\ln p_{ij}=0$ ; the. Calculate the indicator weight vector. $\omega =\left\{\omega_{1},\;\omega_{2},\;\ldots ,\;\omega_{n}\right\}\text{,}$ where $\omega_{j}=(1-S_{j)}/\sum_{j=1}^{m}\left(1-S_{j}\right)$ , and $0\leq \omega_{j}\leq 1,\sum_{j=1}^{m}\omega_{j}=1\text{.}$ After standardizing the data, we utilized the SPSS software to carry out step-by-step processing according to the entropy weight method formula. This resulted in the weights of the 15 indicators. Then, based on the risk composition shown in Table 2, we calculated the weighted values to obtain the specific values of the four types of risks for the 437 1 km*1 km areas in the research region.

#### 2.3.5 K-means algorithm

This study uses the K-means algorithm (42) for clustering to develop prophylactic measures in the analysis of risk. Utilizing the entropy power method, we calculated four distinct types of risk values for 437 grid, each measuring 1 km * 1 km. Subsequently, we employed the SPSS software to perform k-means clustering on these data. The four types of risks were treated as eigenvalues, and based on their compositions, we classified 437 grid into four different categories. This classification allowed us to effectively map out the risk landscape across the various areas.

The K-means algorithm is based on the principle of similarity, which classifies data objects with high similarity into clusters of the same class and those with high dissimilarity into clusters of different classes. The biggest difference between clustering and classification is that the clustering process is an unsupervised process, and the data objects to be processed do not have any prior knowledge. The classification process is a supervised process, and there is a training data set with prior knowledge (43). K-means in the k-means algorithm represents the number of class clusters, and represents the mean value of the data objects within the class clusters (this mean value is a description of the center of the class clusters). Therefore, the K-means algorithm is a partition-based clustering algorithm that uses distance as a measure of similarity between data objects. The smaller the distance between data objects, the higher their similarity, and the more likely they are in the same cluster. There are many types of calculations for the distance between data objects (44). The k-means algorithm usually uses the Euclidean distance to calculate the distance between data objects. Its formula is as follows:


(9) $$R^{2}=\frac{\sum_{z=1}^{n_{c}}\sum_{j=1}^{n_{i}}\sum_{k=1}^{n_{v}}{\left(V_{ij}^{k}-\bar{v_{k}}\right)}^{2}}{\sum_{\dot{r}=1}^{n_{c}}\sum_{j=1}^{n_{i}}\sum_{k=1}^{n_{v}}{\left(V_{ij}^{k}-\bar{v_{t}^{k}}\right)}^{2}}$$

Among them,

*n* = number of features,

$n_{i}$ = number of features in cluster *i*,

$n_{ci}$ = number of clusters,

$n_{v}$ = number of variables used for feature clustering,

$v_{ij}^{k}$ = *k*th variable of the jth feature in the *i*th cluster,

$v^{k}$ = mean of the *k*th variable,

$v_{t}^{k}$ = mean of the *k*th variable in the ith variable.

## 3 Results

### 3.1 Index contribution rate analysis based on random forest model

#### 3.1.1 Ranking and analysis of contribution rate of random forest model results

1. Indicators in descending order of contribution: Road Network Density (RND), Shopping Mall Density (SMD), Public Transport Density (PTD), Educational Facility Density (EFD), Bank Density (BD), Floor Area Ration (FAR), Poi Functional Mix (POI FM), Designated Hospital Distance (DHD), Poverty Level (PL), Average housing price (AHP), Proportion of Aged Population (POAP), Tertiary Hospital Density (THD), Open Space Density (OSD), Night-time Light Intensity (NLI), and Number of Beds Available in Designated Hospitals (NBADH).
2. Among these indicators, the three with contribution rates over 0.1 are Road Network Density (RND), Shopping Mall Density (SMD), Public Transport Density (PTD), indicating that infection risk is most closely associated with these three indications. There were seven indicators with contribution rates between 0.04 and 0.1, including Educational Facility Density (EFD), Bank Density (BD), Floor Area Ration (FAR), Poi Functional Mix (POI FM), Designated Hospital Distance (DHD), Poverty Level (PL), Average housing price (AHP). The Road Network Density (RND), Shopping Mall Density (SMD), and Public Transport Density (PTD), these three indicators are the first, second, and third contributions, respectively, fully reflects the spatial coupling of urban population distribution and activities with public health outbreaks.
3. There are five indicators with contribution rates lower than 0.04, including Proportion of Aged Population (POAP), Tertiary Hospital Density (THD), Open Space Density (OSD), Night-time Light Intensity (NLI), and Number of Beds Available in Designated Hospitals (NBADH). This indicates a weak correlation between these indicator factors and the occurrence of COVID-19 public health emergency infections in the main urban area of Wuhan City.

#### 3.1.2 Optimized selection of evaluation indicators

##### 3.1.2.1 AUC and ACC-related concepts

The Area Under Curve (AUC) (45) is usually used to represent the area under the Receiver Operating Characteristic (ROC) curve and the coordinate axis, and its value is usually in the range of 0.5–1. ACC (Accuracy), which is the proportion of “correct” samples to all predicted samples, is usually used to judge the classification results of a classifier. The combination of AUC and ACC is a measure of the stability and accuracy of the prediction model, which can effectively avoid errors caused by skewed data in actual data samples.

##### 3.1.2.2 Optimized selection of evaluation indexes

For the ranking results of the contribution rate of each indicator, the factors were gradually streamlined, starting from the lowest contributing indicator, and each streamlined indicator was constructed into a new prediction model. The training set, validation set, and model parameters were kept unchanged. Each random forest model was run in turn using Python software, and the AUC and ACC values of each model were recorded and compared to explore the optimal model structure based on the above 2000 samples starting with the indicator with the lowest contribution rate, the indicators were deleted, and the accuracy and stability of the model were judged by comparing the AUC and ACC values of the random forest model to determine the optimal model, as shown in Table 3.

**Table 3:** Experimental procedure record.

Experiment number	Delete indicator	AUC	ACC
0	--	0.913	0.924
1	NBADH	0.911	0.921
2	NBADH,NLI	0.908	0.909
3	NBADH,NLI,OSD	0.911	0.923
4	NBADH,NLI,OSD,THD	0.915	0.923
5	NBADH,NLI,OSD,THD,POAP	0.920	0.927
6	NBADH,NLI,OSD,THD,POAP,AHP	0.899	0.911
7	NBADH,NLI,OSD,THD,POAP,AHP,PL	0.899	0.906
8	NBADH,NLI,OSD,THD,POAP,AHP,PL,DHD	0.881	0.894
9	NBADH,NLI,OSD,THD,POAP,AHP,PL,DHD,POIFM	0.87	0.886

As evidenced by Table 1, the random forest model, which incorporates 15 indicators, demonstrates high precision with AUC and ACC values exceeding 0.9. Based on the evaluation metrics, Experiment 5, which eliminated five indicators, achieved the highest level of accuracy. The five indicators that were removed are: Proportion of Aged Population (POAP), Tertiary Hospital Density (THD), Open Space Density (OSD), Night-time Light Intensity (NLI), Number of Beds Available in Designated Hospitals (NBADH). The five most prominent indicators contributing to the risk of COVID-19 epidemic in Wuhan were the Road Network Density (RND), Shopping Mall Density (SMD), Public Transport Density (PTD), Educational Facility Density (EFD), Bank Density (BD). Floor Area Ration (FAR), Poi Functional Mix (POI FM), Designated Hospital Distance (DHD), Poverty Level (PL), and Average housing price (AHP) also contributed significantly to the risk assessment of the epidemic.

### 3.2 Four types of risk composition and result analysis

#### 3.2.1 Determining the weight of each index using the entropy weighting method

Utilizing the entropy weight method, 15 evaluation indicators were employed as secondary indicators, and four types of risks were designated as primary indicators. The weights of each indicator, which constitute the four types of risks, were calculated to construct a risk assessment model for the COVID-19 public health emergency in Wuhan. To overcome the errors caused by human subjectivity and differences in data attributes on the prediction results. An entropy weighting method was introduced for index weighting. The model is constructed iteratively to maximize its prediction accuracy and calculate weights of each evaluation index relative to the final risk based on prediction errors in each round. The entropy weighting method was executed using Python software to obtain the consequences of each evaluation index (Table 4).

**Table 4:** Entropy weighting method to determine the weights of each index.

Indicator name	Weight of each indicator
Number of Beds Available in Designated Hospitals (NBADH)	0.061131789
Open Space Density (OSD)	0.061316449
Educational Facilities Density (EFD)	0.062547197
Bank Density (BD)	0.062568905
Poverty Level (PL)	0.062573166
Road Network Density (RND)	0.062589868
Shop Mall Density (SMD)	0.062590588
Tertiary Hospital Density (THD)	0.062597494
Number of Infected Cases (NIC)	0.06264185
Public Transport Density (PTD)	0.062656595
Floor Area Ration (FAR)	0.062734661
Average Housing Price (AHP)	0.062743014
Designated Hospital Distance (DHD)	0.062763455
Percentage of Aged Population (POAP)	0.062835191
Poi Functional Mix (POI FM)	0.062849327
Night-time Light Intensity (NLI)	0.062860452

The standardized data from the 15 categories of indicators were utilized as secondary indicators. Each of these was multiplied by its respective weight, and then combined with the composition of the four types of risks as outlined in Table 2. The sum of these calculations yielded four primary indicators, which represent the specific values of the four types of risks.

#### 3.2.2 Analysis of risk results

**Figure 4:**
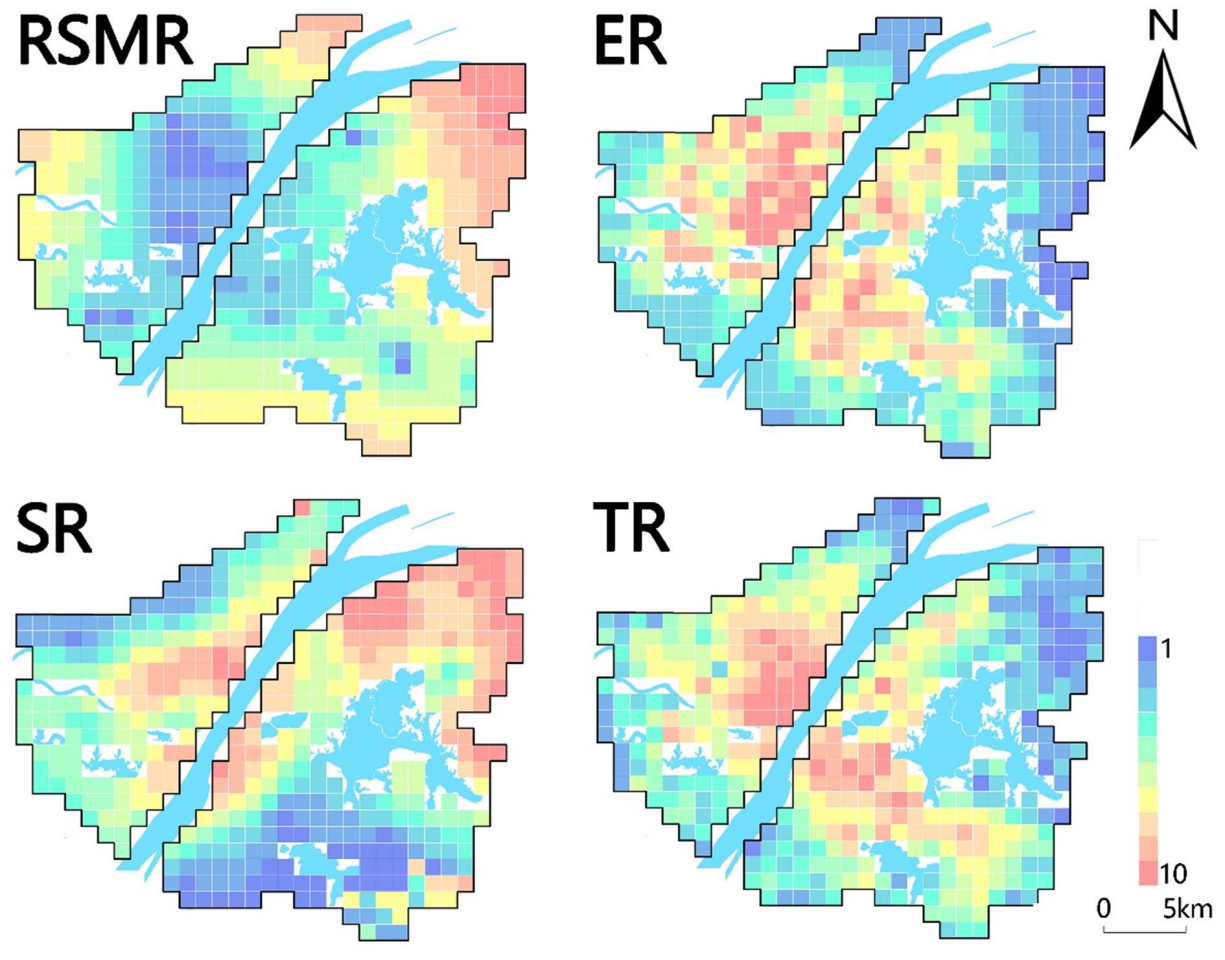
Schematic diagram of four types of risks.

After determining the specific values of the four types of risks using the entropy weight method, we utilized ArcGIS software to associate these risk values with 437 grids, each measuring 1 km by 1 km, within the study area. This procedure led to the generation of four distinct risk maps, as depicted in Figure 4. Each type of risk distribution presents unique characteristics, and the analysis was carried out in line with the specific conditions of the central urban area of Wuhan.

1. Exposure risk refers to the likelihood of coming into contact with the virus and possibly becoming infected. It is determined by factors that affect the chances of transmission from an infected person to a susceptible person. Understanding the risk of exposure is essential for making informed decisions about activities, precautions, and public health measures to prevent the spread of the virus. A 1 km × 1 km buffer zone was created for the case points to estimate the exposure level of the virus. In addition, night-time light intensity data within the study area were used to estimate the urban compact spatial pattern and population aggregation intensity within the grid. Related studies have shown that the higher the night-time light intensity, the higher the risk of exposure in places with an urban compact spatial pattern or population aggregation intensity. This study combines these two parameters to assign weights for comprehensive analysis of exposure risk.
  As shown in Figure 4 (ER), four sites in the study area were at a high risk of exposure: Jianghan District East, Wuchang District South, Optical Valley, and Qingshan District East. Qiaokou District and Wuchang District are highly developed economically therefore movement of people is inevitable. As a result, these districts become high-exposure-risk areas. Qingshan District has a poor economy and the highest exposure risk. This is probably due to the large number of people leaving Wuhan at the beginning of the epidemic, which led to a surge in the flow of people at Wuhan Station in Qingshan District, accelerated the spread of the virus, and resulted in a sharp increase in the number of infections. Hankou and Wuchang Stations had a large number of cases, consistent with the prediction. Overall, with high levels of economic development and risk in regions with high human mobility. In contrast, viral exposure is a prerequisite for viral transmission, which is faster in places with high exposure risk.
2. Transmission risk, denoting the likelihood of virus spread from an infected to a susceptible individual, holds critical significance for the implementation of effective public health measures aimed at controlling and preventing disease transmission. This research employed various factors, including open-space density, road network density, educational facility density, public transport density, and average housing prices, to assess transmission risk profiles. Open spaces, due to high population movement and crowding effects, were scrutinized. Additionally, spatial metrics such as road network density and public transport density were analyzed to gauge the compactness of transportation infrastructures and indirectly infer population migration intensity within the city. Weightage was assigned to each parameter, enabling a comprehensive analysis of propagation risk.
  Transmission risk, as illustrated in Figure 4 (TR), primarily signifies human contact and mobility intensity. The central region of Hankou, the most economically advanced area in Wuhan with extensive construction and comprehensive public amenities, exhibited the highest transmission risk. Wuchang District, the second-largest economic development zone in Wuhan, also displayed elevated transmission risk. Optical valleys, buoyed by government support and the influx of enterprises, presented heightened transmission risk compared to neighboring regions. Conversely, areas with lower transmission risk were typically situated at the periphery of the study area. These regions exhibited limited economic development and infrastructure, leading to reduced economic activities and human interactions.
3. Risk of Scarcity of Medical Resources for the purpose of analyzing the abundance of medical resources in the study area. The lack of healthcare resources is a major public health problem, and it is believed that the COVID-19 outbreak will exacerbate the negative effects of the pandemic by straining healthcare resources. Not only will those infected by the COVID-19 be affected, but patients with other diseases will also be affected by the crowding of medical resources caused by the COVID-19, resulting in delayed treatment and possibly death. In this context, the risk of healthcare scarcity is a measure of the availability and adequacy of resources for the effective functioning of healthcare facilities. In this study, we used the location and bed data of designated hospitals established by the Health Inspection Commission during the epidemic in Wuhan, and the huff gravity model to measure the risk of medical resource scarcity.
  The risk of medical resource scarcity was analyzed in terms of the abundance of medical resources in the study area. According to (RSMR) in Figure 4, it can be seen that the places with a high risk of medical resources are located in the periphery of the city, and such areas are far from the designated hospitals. Thus, the spread of the pandemic may have been exacerbated. However, in well-developed areas, there are often better medical resources closer to designated hospitals and a lower risk of scarcity of medical resources. If the new crown pandemic spreads again in Wuhan, medical resources in the peripheral areas of the city will be rapidly depleted and medical functions will subsequently be reduced. In such areas, complete medical resources and quarantine equipment, such as drugs, respirators, protective clothing, and masks, should be prioritized.
4. Susceptibility risk refers to the likelihood of individuals contracting the COVID-19 virus, with the older adult being particularly vulnerable. Research indicates that while many COVID-19 cases result in mild symptoms and recovery, older adults are disproportionately affected, with a higher mortality rate. Data from the Centers for Disease Control and Prevention (CDC) in October 2021 revealed that 76% of COVID-19-related deaths in the United States occurred in individuals over 65 years old. Additionally, susceptibility risk is influenced by poverty levels, as impoverished areas often lack adequate medical and protective resources, amplifying the impact on vulnerable populations. This study assessed susceptibility risk using empirical Bayesian kriging interpolation for age demographics and GDP data from Wuhan City.
  Areas with high susceptibility risk have a greater likelihood of infection post-exposure, leading to severe illness or death. The old city of Wuhan exhibited the highest susceptibility risk (Figure 4; SR), attributed to its dense local and external population, with a significant older adult demographic. Qingshan District, a central industrial town, had elevated susceptibility due to its aging workforce and economic factors, notably the presence of major industrial enterprises. Given the current relatively relaxed epidemic prevention policies, it is crucial for society to focus on susceptibility risks, emphasizing the safety of older adult and young populations.

### 3.3 Cluster analysis and prevention strategies

**Figure 5:**
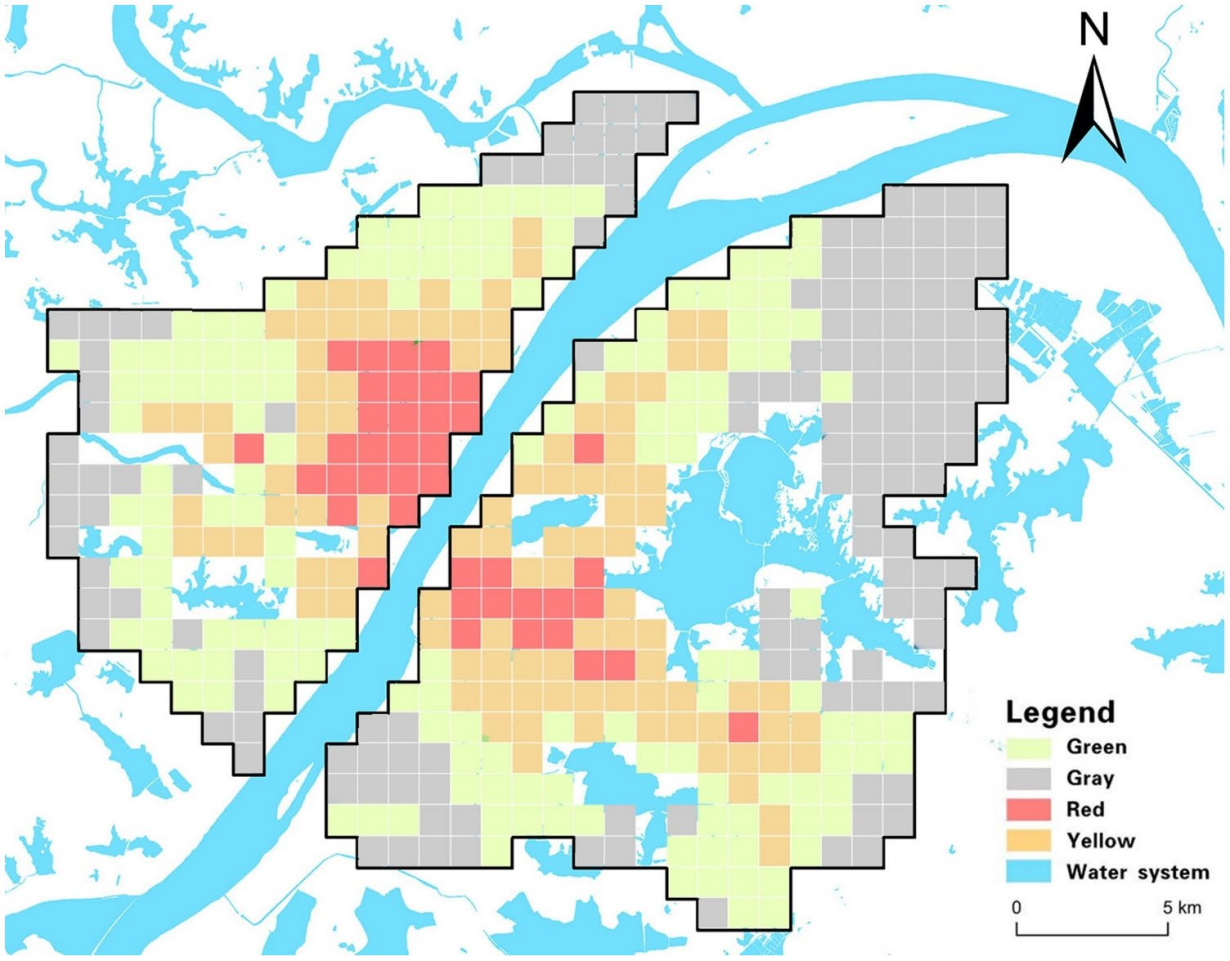
Risk clustering diagram.

**Figure 6:**
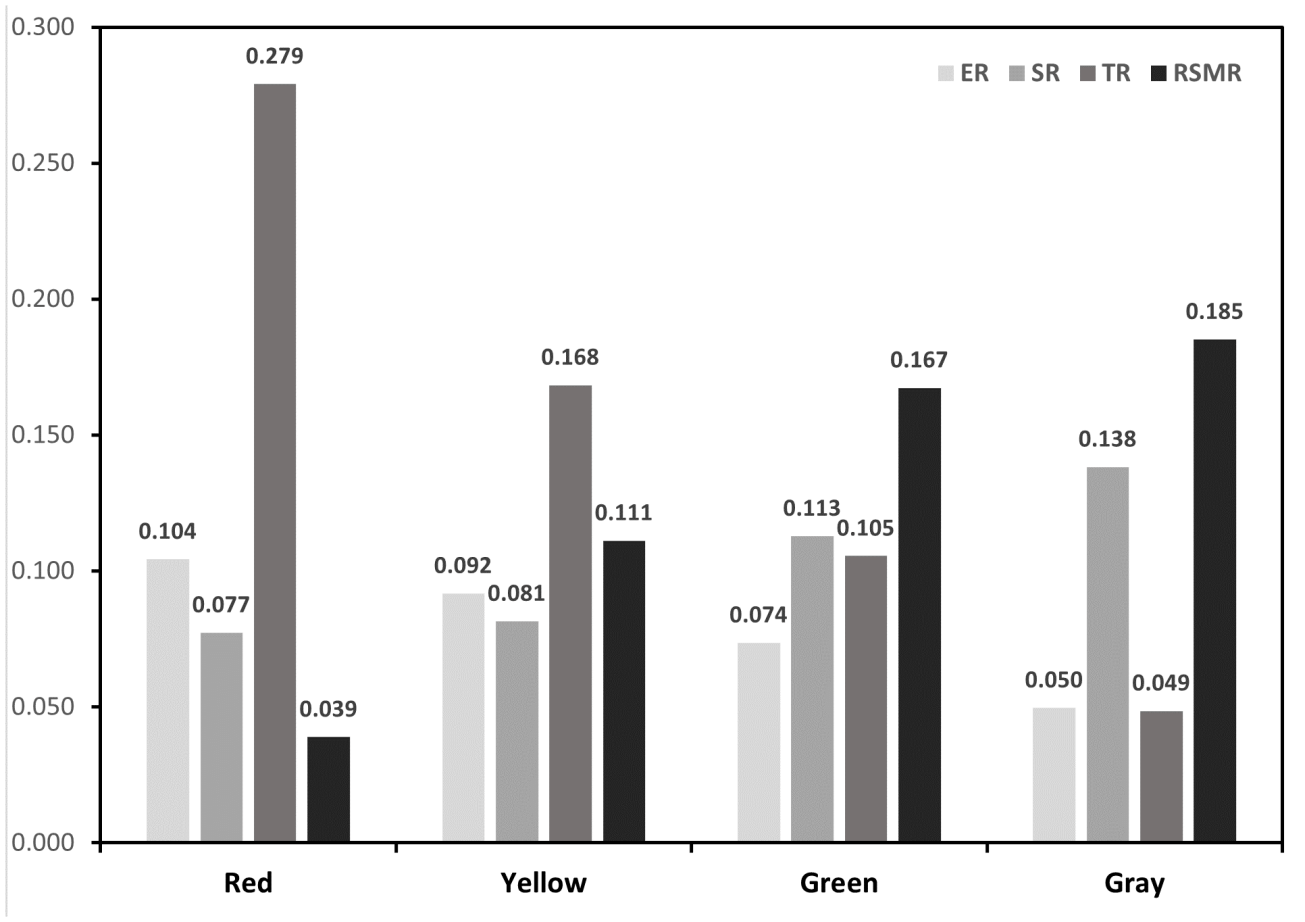
Four clustering features.

To formulate targeted epidemic prevention strategies, a comprehensive analysis synthesizing various risk factors related to COVID-19 was conducted. This analysis encompassed transmission, exposure, susceptibility, and medical resource scarcity risks. Utilizing a multivariate clustering approach and the K-means algorithm, Wuhan’s main urban area was categorized into four distinct regions with similar risk profiles: red zone, yellow zone, green zone, and gray zone. The resulting risk groups are illustrated in Figure 5, depicting the risk distribution across the city’s main urban area. Additionally, Figure 6 provides specific values for the center points of each risk cluster in individual subdistricts. By scrutinizing the risk composition of these central points, representative characteristics of the combined risk types in each region were identified, enabling precise assessments for corresponding areas and facilitating the development of accurate preventive measures.

1. The red zone, situated primarily within Wuhan’s core area and encompassing locations like Jiedaokou Street and Jianghan Street, faces the highest risks in exposure and transmission, ranking third in susceptibility risk while having the lowest risk of medical resource scarcity. This zone is characterized by high population density, extensive infrastructure, and a bustling economy. Its strategic location within the first ring road of Wuhan and the concentration of service centers make it a hub for numerous people and tourists. However, the red zone benefits from a surplus of medical resources, notably tertiary hospitals (as indicated by THD in Figure 2), which significantly reduces the risk of medical resource scarcity. Given the ample medical resources in this region, targeted strategies should leverage these facilities to control potential outbreaks effectively. Swiftly admitting positive patients upon outbreak detection can mitigate rapid spread, stemming from high exposure and transmission risks. This approach prevents the widespread dissemination of the outbreak across regions through human movement, capitalizing on the area’s robust healthcare infrastructure.
2. In the yellow zone, exposure risk, transmission risk, and susceptibility risk all rank second, while the risk of medical resource scarcity is third. This area, mainly within Wuhan’s second ring road and the optical valley region, is characterized by a dense road network, a concentration of educational institutions and businesses, and high-density public transportation. It includes residential spaces for the city’s working population, major enterprises, and a relatively young demographic.
  To address these specific risks, targeted strategies in the yellow zone should concentrate on managing high-density public transportation effectively. Implementing hierarchical management and control within enterprises, along with school-based measures for students, can significantly reduce the risk of transmission and exposure during outbreaks. Precise prevention measures must focus on restricting crowd gathering activities and limiting the movement of infected individuals in and out of hospitals, schools, and other densely populated areas. Implementing precise regional closures and other measures can protect vulnerable populations, especially those with underlying health conditions, from potential exposure. This tailored approach ensures that the unique characteristics of the yellow zone are considered in developing effective prevention and control measures.
3. The exposure risk, transmission risk and susceptibility risk of the green zone are the third, and the risk of scarcity of medical resources is the second. The green zone is characterized by relatively low population density and scarcity of medical resources. The green zone is located in the peripheral area of the central risk area, which is closer to the urban center. Most of the area is within the third ring road. Whether to slow down the spread of the virus, will be the key to virus control. The targeted strategy for this region should be based on the scarcity of medical resources and the prevention and control units based on residential areas. Such areas should provide medical assistance through increased medical training of community personnel, if necessary. Social counseling measures are also critical to reducing the proportion of severe illness and risk of transmission in susceptible populations in this area.
4. Exposure risk and transmission risk in the gray zone are the fourth, susceptibility risk, and the highest risk of scarcity of medical resources. This area is characterized by a small population, serious aging and far away from the main urban area, with few supporting services in the city. The focus of targeted strategies in this region should be to protect susceptible groups and make medical emergency reserves. Such areas need to be isolated to the greatest extent possible; therefore, relevant settings, such as nursing homes and kindergartens, need to have strict isolation facilities to prevent imported cases. The rapid spread of the virus to the gray area indicates that the virus is expanding in an uncontrollable manner and is most likely spreading to other administrative regions and even neighboring cities around Wuhan.

## 4 Discussion

Due to data accuracy considerations, the primary research area in this study primarily consists of 1 km*1 km grids. However, at this scale, it is insufficient for a nuanced examination of epidemic risk at the micro-level. By adopting the block functional area as the fundamental unit of analysis and integrating its three-dimensional structure with demographic composition, a more precise analysis can be conducted. The inclusion of detailed urban block morphology in the study would contribute to a more comprehensive understanding of the interplay between urban form and epidemic risk.

Owing to data limitations, the formulation of targeted policies did not take into account grassroots public service points in each partition, despite their significant role during the epidemic. It is advisable to explore the full utilization of public service facilities in each region, thereby refining the granularity of targeted policies. Additionally, an investigation into the influencing factors of COVID-19 cases is warranted. The incorporation of alternative model algorithms for comparative analysis would contribute to a more comprehensive assessment.

During the epidemic, the acquisition of location data for Weibo help-seeking cases allowed for the integration of urban environmental and economic data, enabling the analysis of various risk characteristics. As urban multi-dimensional data becomes increasingly abundant, corresponding risk indicators can be further enhanced in terms of completeness and accuracy. It is noteworthy that this study places a significant emphasis on exploring the delicate equilibrium between epidemic control and economic development. Using this as a point of departure, distinct strategies are proposed based on the disparities in risk composition across different partitions.

The study clustered four types of risks resulting in four partitions, and targeted policies were formulated based on the composition of these risk types within each partition. This approach, compared to singular analysis of COVID-19 impact factors, holds significant practical importance. Similar methodologies can be applied in future public health events to swiftly discern unique risk profiles across partitions, facilitating the rapid formulation of targeted policies. These policies aim to uphold normal social development while balancing prevention and control measures with economic development efforts.

## 5 Conclusion

To explore the influence of the urban environment on the spread of the epidemic during public health emergencies in Wuhan, we combined the random forest model and entropy power method to investigate the factors influencing public health emergencies and the risk assessment of epidemic spread. The main conclusions are as follows.

1. The study identified the five most influential indicators contributing to the risk of the Wuhan COVID-19 outbreak, which include Road Network Density (RND), Shopping Mall Density (SMD), Public Transport Density (PTD), Educational Facility Density (EFD), and Bank Density (BD). Additionally, Floor Area Ratio (FAR) and Point of Interest Functional Mix (POI FM) were considered.
2. By eliminating less influential indicators, including the Proportion of Aged Population (POAP), Tertiary Hospital Density (THD), Open Space Density (OSD), Night-time Light Intensity (NLI), and Number of Beds Available in Designated Hospitals (NBADH), the random forest model attained the highest level of predictive accuracy in assessing COVID-19 risk.
3. The spatial characteristics of the four categories of COVID-19 epidemic risk, namely transmission risk, exposure risk, susceptibility risk, and Risk of Scarcity of Medical Resources, exhibited significant differentiation. These characteristics were utilized to establish a four-level integrated risk zoning system through K-MEANS clustering. The distribution pattern of these zones adhered to a “multicenter-periphery” gradient diffusion. This zoning system, when integrated with the inherent characteristics of the urban environment within distinct zones, allows for the customization of control strategies tailored to the respective risk compositions.

This study findings not only highlight the key risk indicators associated with the Wuhan COVID-19 outbreak but also underscore the importance of refining modeling techniques for improved predictive accuracy. Furthermore, the spatial analysis of epidemic risk categories and the subsequent development of differentiated control strategies hold practical implications for managing future public health crises.

## Data availability statement

The original contributions presented in the study are included in the article/supplementary material, further inquiries can be directed to the corresponding author.

## Author contributions

PZ and LL: conceptualization. PZ: methodology and project administration. HZ: software. HZ and ZC: validation YP: formal analysis and investigation. XS: resources. CL: data curation. HZ: writing—original draft preparation, writing—review and editing, and visualization. LL: supervision. All authors have read and agreed to the published version of the manuscript.
